# Natural compounds solasonine and alisol B23-acetate target GLI3 signaling to block oncogenesis in MED12-altered breast cancer

**DOI:** 10.22099/mbrc.2024.49044.1915

**Published:** 2024

**Authors:** Shivani Akula, Cristian G. Gonzalez, Sophia Kermet, Marieke Burleson

**Affiliations:** 1 Department of Chemistry and Biochemistry, University of the Incarnate Word, San Antonio, TX, USA; 2 Department of Biology, University of the Incarnate Word, San Antonio, TX, USA; † These authors contributed equally to this work

**Keywords:** MED12, GLI3, breast cancer, solasonine, alisol B23-acetate

## Abstract

Breast cancer remains to be the second leading cause of cancer deaths worldwide thereby highlighting the critical need to find superior treatment strategies for this disease. In the current era of cancer treatment, personalized medicine is garnering much attention as this type of treatment is more selective thereby minimizing harmful side effects. Personalized medicine is dependent upon knowing the underlying genetic landscape of the initial tumor. In our study, we focused our efforts on a specific subset of breast cancer that harbors genetic alterations in the Mediator subunit 12 (MED12). Our results show that loss of MED12 leads to enhanced cellular proliferation and colony formation of breast cancer cells through a mechanism that involves activation of GLI3-dependent SHH signaling, a pathway that is central to breast development and homeostasis. To find a personalized treatment option for this subset of breast cancer, we employed a natural compound screening strategy which uncovered a total of ten compounds that selectively target MED12 knockdown breast cancer cells. Our results show that two of these ten compounds, solasonine and alisol B23-acetate, block GLI3-dependent SHH signaling which leads to a reversal of enhanced cellular proliferation and colony formation ability. Thus, our findings provide promising insight into a novel personalized treatment strategy for patients suffering from MED12-altered breast cancer.

## Introduction

Improved treatment strategies for breast cancer are of critical importance as this disease remains the second leading cause of cancer deaths among women [1]. Chemotherapy and radiation are among the frontline choices of treatment for breast cancer, however, there are discernable issues regarding these methods. First, these treatments are well known to have detrimental side effects for the patient, such as fatigue, extreme nausea, and infection. Second, it is well established that different subtypes of cancer, including breast cancer, display differential responses to generic treatment options [2-4]. Thus, there is a critical need to discover personalized treatment options for specific subsets of breast cancer that not only eliminate detrimental side effects but also show an improved treatment response. Breast cancer initiation and progression is mainly dependent upon changes in the genetic profile of an individual as defined by either gene expression alterations or gene mutations. Several of these genetic alterations have been uncovered over the years, including upregulation or gene amplification of the estrogen receptor, the progesterone receptor, or the HER2 gene [5]. More recently, however, mediator subunit 12 (MED12) has been identified as an additional genetic alteration that presents itself in up to 33% of breast cancers [6-8]. Though this indicates that MED12 likely plays an important role in breast cancer oncogenesis there currently is very limited knowledge on the mechanism of action behind MED12-mediated breast cancer. 

MED12 is a subunit of Mediator, a large multi-subunit complex that plays critical roles in gene regulation. MED12 has been shown to regulate Mediator function by serving as a bridge between gene-specific transcription factors and RNA polymerase II [9, 10]. As a result of these physical interactions, MED12 plays a direct role in activating and repressing transcription of target genes. Previous studies have shown that either, mutations within, or loss of MED12 can promote structural changes within Mediator to disrupt gene regulation [11-13]. Importantly, GLI3, a downstream regulator of Sonic Hedgehog (SHH) signaling, is among these dysregulated genes [14, 15]. Dysregulated GLI3-dependent SHH signaling, as a result of mutant or downregulated MED12, has been shown to play an important role in X-linked disability syndromes and prostate cancer progression [15, 16]. These findings are of high importance as SHH signaling, a stromal-epithelial pathway, is also central to breast development and homeostasis and has previously been shown to play a key role in breast cancer tumorigenesis [17, 18]. Thus, we hypothesize that alterations in MED12 causes dysregulation of SHH signaling in breast cancer cells to subsequently promote oncogenesis. 

Drug development studies have indicated that an estimated 60% of future anti-cancer agents will be from natural origin [19]. Many natural compounds have already proven to be highly effective in treating cancer either independently or in a synergistic effect with chemotherapeutic agents [19, 20]. Importantly, the use of natural compounds drastically reduces the side effects that occur when using chemotherapeutic agents alone [19, 20]. Currently, studies that investigate natural compounds specifically for MED12-altered breast cancer are lacking; therefore, in this study, we have utilized the DiscoveryProbe natural compound library to screen natural compounds for their anti-cancer activity on breast cancer cells that have a down-regulation of MED12 expression.

## MATERIALS AND METHODS

**Cell culture: **MCF-7 control and MCF-7 MED12 knockdown cells were regularly cultured at 37°C and 5% CO_2 _in Dulbecco’s Modified Eagle Medium (DMEM) supplemented with 10% fetal bovine serum (ThermoFisher) and penicillin-streptomycin-L-glutamine (Invitrogen). 

**Generation of MED12 knockdown cell line: **pLKO.1 vector containing no shRNA (shCT) or MED12 shRNA (shMED12) were transfected into HEK293T cells together with psPAX2 and pMD2.G (Invitrogen). Viral supernatants were harvested for infection of MCF-7 cells and infected cells were selected with 1 µg/ml puromycin. 

**Treatment of cells with solasonine, AB23A, or patchouli alcohol: **For drug treatment, cells were seeded at optimized concentrations in 96-well, 24-well, 6-well, 60 mm, or 100 mm plates. One day post-seeding, media was removed and replaced with media containing 1 µM drug or equal volume amount of DMSO. 

**Quantitative real time PCR: **Cells were seeded at 5×10^5^ cells in 60mm dishes and RNA was extracted two days later using Trizol reagent. RNA was reverse transcribed using oligo(Dt) and superscript III (Invitrogen) following standard procedures and used for quantitative PCR. For drug-treated cells, cells were seeded at 4×10^5^ cells in 60mm dishes on day one, treated with 1 µM drug on day two, and harvested for quantitative PCR as described above on day four. Primer sequences used in quantitative PCR are as follows: GLI1 (forward: primer: TTTGGACCCAACTTGCCCAA and reverse primer: GCCCTATGTGAAGCCCTATT), ASCLI (forward primer: AAGAGCAACTGGGACCTGAGTCAA and reverse primer: AGCA AGAACTTTCAGCTGTGCGTG), CREB5 (forward primer: CGTGCCTCCTTGAAACAAGC CATT and reverse primer: ATGAAACACCAGCACCTGCCTAGA), NGN2 (forward primer: AGGGCAGGTGTAGCCTTTCTGATT and reverse primer: CGCCACCCTTGGCTTTGACA ATAA).

**Western Blot: **For western blot analysis, whole cell lysates were prepared and 100 µg total protein was resolved on 10% SDS-PAGE. Protein was detected using the following antibodies at indicated dilution: anti-MED12 (Invitrogen PA5-51852 @ 1:1000 dilution) and anti-TFIIEβ (Invitrogen 11596-1-AP @ 1:3000 dilution).

**Proliferation assays: **Cells were seeded at 1×10^4^ cells per well in 24-well plates in triplet repeats in 2 ml DMEM. Cells were harvested on indicated days using standard trypsin protocols and counted on a hemocytometer. For drug-treated cells, media was removed one day after seeding and replaced with DMEM containing either DMSO or 1 µM drug. 

**MTT assays: **Cells were seeded at 1×10^4^ cells per well in 96 well plates in triplet repeats in 100 ml DMEM. The following day media was replaced with DMEM containing either DMSO or 1 µM drug. Two days later, MTT assays were performed using the CyQUANT MTT Viability Assay (ThermoFisher) following standard procedures. 

**Colony formation assays: **Cells were seeded at 4000 cells/well in a 6-well plate using in 2 ml DMEM. One day after seeding, media was removed and replaced with DMEM containing either DMSO or 1 µM drug. Media was changed every 3 days. On day 21, cells were washed with PBS, fixed with 10% paraformaldehyde and stained with 0.1% crystal violet.

**Figure 1:**
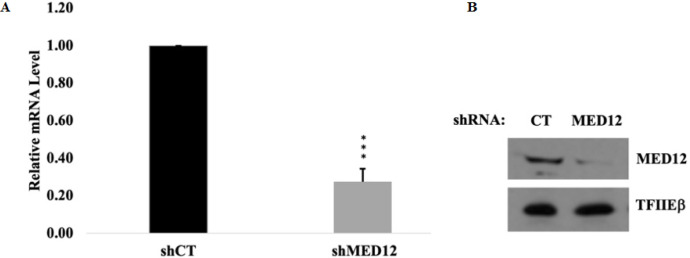
Confirmation of MED12 knockdown in MCF-7 breast cancer cells.

**Figure 2:**
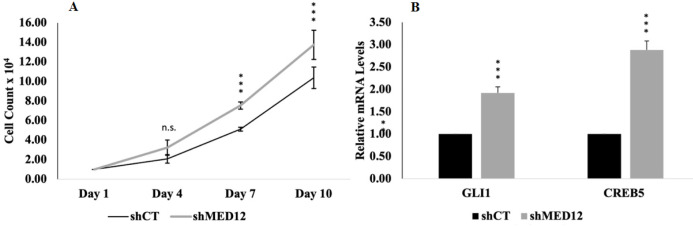
MED12 knockdown promotes enhanced cell proliferation and hyperactivated GLI3-dependent SHH signaling.

**Quantitative analysis: **All statistical analyses were performed using the standard type 2, two-tail T-test. A cutoff P value of 0.05 was used to indicate significance. For figures 1 and 2, shMED12 results were compared to shCT results to determine significance. In figure 4 and 5, drug-treated results were compared to DMSO-treated results to determine significance. 

## Results

To study the role of MED12 in breast cancer, we first generated a stable MED12 knockdown MCF-7 cell line through lentiviral-mediated shRNA delivery. Since tumor-associated MED12 mutations lead to full inactivation of protein function, our knockdown system should recapitulate a MED12 mutant setting. Knockdown efficiency was assessed through quantitative PCR and western blot analysis (Fig. 1a, b). 

Next, proliferation assays were performed on our MED12 knockdown cells to confirm that loss of MED12 enhances breast cancer oncogenesis (Fig. 2a). Since prior results have shown a link between loss of MED12 and hyperactivation of GLI3-mediated SHH signaling in prostate cancer and X-linked intellectual disability syndromes, we performed quantitative PCR to determine whether dysregulated signaling also occurs in breast cancer cells. Our results confirmed that loss of MED12 promotes the expression of GLI3 target genes (Fig. 2b). 

A) Confirmation of MED12 knockdown in MCF-7 cell by qRT-PCR using primers against MED12. mRNA levels are normalized against GAPDH and expressed relative to the levels in cells infected with pLKO.1 vector containing no shRNA (shCT). Data is mean ± SEM from 4 replicate assays. B) Confirmation of MED12 knockdown via western blot analysis. (Unpaired two-tailed student t-test: ***P<0.0001).

A) Proliferation assays of MED12 knockdown (shMED12) and control (shCT) cells. Data is mean ± SEM from 3 replicate assays. B) qRT-PCR of shCT and shMED12 cells using primers against the GLI3 target genes, GLI1 and CREB5. mRNA levels are normalized to GAPDH and expressed relative to their levels in shCT cells. Data is mean ± SEM from 3 replicate assays. (Unpaired two-tailed student t-test: ***P<0.0001, n.s: not significant).

**Figure 3:**
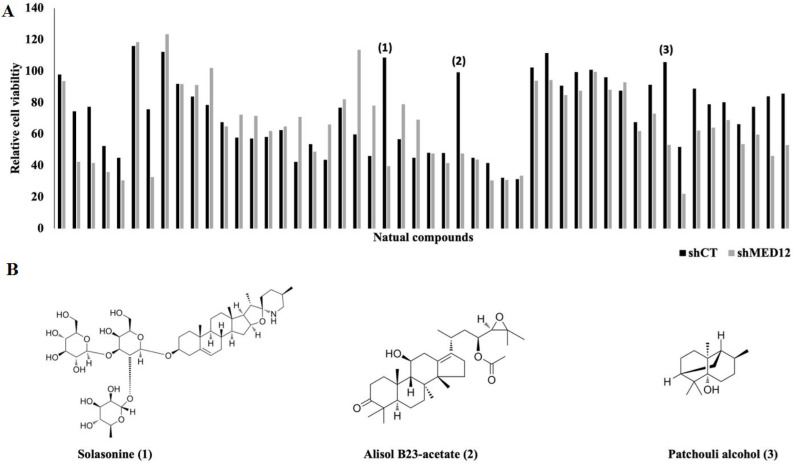
MTT viability assay from DiscoveryProbe natural compound screen.

Since targeted therapy is rapidly rising as a superior treatment option for cancer patients, we next set out to uncover a novel treatment strategy for MED12 downregulated breast cancer. We used the DiscoveryProbe natural compound library to screen a total of 49 compounds for their anti-cancer activity in MED12 knockdown breast cancer cells. shControl and shMED12 MCF-7 cells were treated with either DMSO or 1 µm natural compound for 48 hours, followed by MTT assay to screen for cell viability. Using a cutoff of 20% viability difference, we found a total of 10 compounds that had selective anti-cancer activity against our MED12 knockdown cells (Fig. 3a). We selected the three compounds with the most drastic effect for further analysis, which were identified as Solasonine, Alisol B23-acetate (AB23A), and patchouli alcohol (Fig. 3b). Solasonine is a glycoalkaloid that is found in the Solanum plant. Anti-cancer activity has been shown for solasonine in hepatocellular carcinoma, malignant glioma, non-small cell lung cancer, and digestive system tumors, but no reports have currently shown activity in breast cancer cells [21-23]. AB23A is a natural triterpenoid that has been isolated from the Chinese medicine rhizome alismatis. It has previously been shown to have anti-cancer activity in a variety of cancers, mainly through anti-apoptotic strategies [24-29]. Currently, no link has been reported between AB23A and the SHH signaling pathway nor has AB23A been shown to have anti-cancer activity in breast cancer cells. Patchouli alcohol is a tricyclic sesquiterpene and the dominant bioactive component in oil from Pogostemon cablin. Patchouli alcohol has previously been reported to have anti-cancer activity in a variety of cancers though no activity has been reported in breast cancer cells [30-35].

A) Quantitative result from MTT viability assay of shCT and shMED12 cells. Cell viability was normalized to shCT cells treated with DMSO. A total of 49 natural compounds were tested. (1), (2), and (3) annotate solasonine, alisol B23-acetate, and patchouli alcohol as shown in (B). B) Chemical structures of solasonine, alisole B23-acetate, and patchouli alcohol.

**Figure 4:**
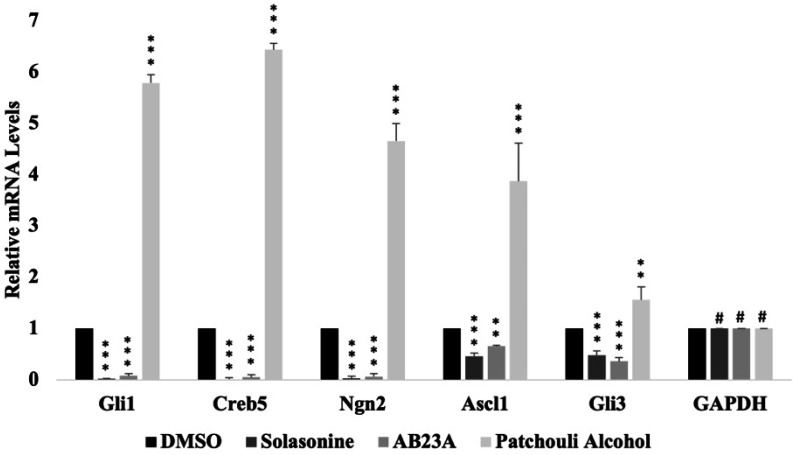
Treatment of MED12 knockdown cells with solasonine or alisol B23-acetate blocks GLI3-dependent SHH signaling.

**Figure 5:**
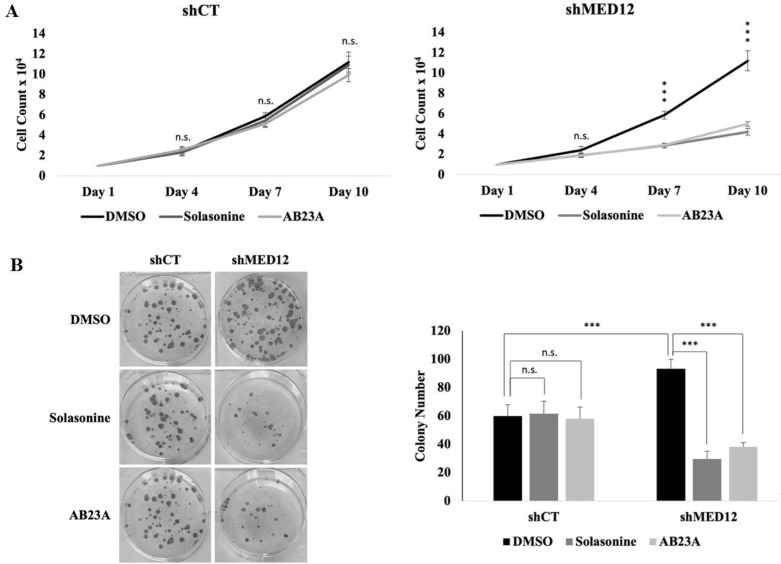
Solasonine and alisol B23-acetate treatment reverses enhanced cell proliferation and colony formation.

Since our MED12 knockdown cells displayed hyperactivated GLI3-dependent SHH signaling, we hypothesized that solasonine, AB23A, and patchouli alcohol potentially target this signaling pathway thereby reversing the oncogenic effects in our knockdown cells. Interestingly, prior studies have already shown that solasonine has an inhibitory function against Gli-mediated transcriptional activity thereby further supporting our hypothesis [36]. Though AB23A and patchouli alcohol have been linked to other signaling pathways to exert anti-cancer effects, no link has been established to SHH signaling. Thus, we performed quantitative PCR on our MED12 knockdown cells that were treated with either DMSO or 1 µM solasonine, AB23A, or patchouli alcohol. Interestingly, we observed that solasonine and AB23A drastically block the expression of GLI3 target genes, whereas patchouli alcohol causes an upregulation of these genes (Fig. 4). Consequently, we focused our subsequent efforts on solasonine and AB23A. To further study the effects of solasonine and AB23A, we performed proliferation and colony formation assays. Both assays showed that treatment with either compound significantly blocks the proliferation and colony formation ability of our MED12 knockdown cells (Fig. 5a,b). Importantly, treatment did not affect our MCF-7 control cells, thus further supporting that solasonine and AB23A selectively target MED12 knockdown cells (Fig. 5a,b). Overall, these results suggest that solasonine and AB23A inhibit GLI3-mediated SHH signaling and are therefore promising therapeutic agents for MED12 downregulated breast cancer. 

qRT-PCR of MED12 knockdown cells treated with DMSO, solasonine, alisol B23-acetate (AB23A), or patchouli alcohol. mRNA levels are normalized to GAPDH and expressed relative to the levels in cells treated with DMSO. Data is mean+/- SEM from 3 replicate assays. (Unpaired two-tailed student t-test: ***P<0.0001, **P<0.001, # non-significant).

A) Proliferation assays of shCT cells (left) or shMED12 cells (right) treated with DMSO, solasine, or alisol B23-acetate. B) Left: Colony formation images of shCT or shMED12 cells treated with DMSO, solasonine, or alisol B230-acetate. Right: quantification of colony formation assay. Data is mean ± SEM from 3 replicate assays. (Unpaired two-tailed student t-test: ***P<0.0001, n.s: not significant) .

## Discussion

In summary, we found that loss of MED12 promotes GLI3-dependent SHH signaling in breast cancer cells which subsequently leads to enhanced cell proliferation and colony formation ability. Furthermore, we successfully found two natural compounds that can potentially combat MED12-altered breast cancer by targeting the GLI3-dependent SHH signaling pathway. Specifically, we found that treatment with solasonine, a glycoalkaloid from the solanum plant, or AB32A, a triterpenoid isolated from the Chinese medicine rhizome alismatis, drastically reduces the expression of GLI3 target genes in our MED12 knockdown cells which subsequently leads to reduced proliferation and colony formation ability of these cells. Importantly, treatment with either compound does not affect our MED12 wildtype cells thus indicating that these compounds have strong selectivity. This is of critical importance as it indicates that the compounds do not target cells with physiological levels of SHH signaling thereby likely reducing the possibility of side effects normally observed with chemotherapeutic agents. To further rule out the possibility of cytotoxic effects in cells with physiological SHH signaling, in vivo studies will need to be carried out. Overall, our results show that solasonine and AB23A are promising targeted therapies for breast cancer cells with dysregulated GLI3-dependent SHH signaling such as is seen in MED12-altered cells. 

In conclusion, our study has provided critical insights into the mechanistic basis behind MED12-altered breast cancer progression and, importantly, how this disease could potentially be combatted. Though prior reports have established links between SHH signaling and breast cancer development, no studies to date have reported the potential role of MED12 in this process [17, 18]. Our results therefore provide novel and critical insight into the progression and potential treatment of a disease that affects a large proportion of women. We are hopeful that with additional in vivo studies the full potential of these drugs as a novel treatment strategy will be realized. 

## Conflict of Interest:

The authors declare no conflicts of interest.

## Authors’ Contribution:

SA, and CG contributed eqully to the experiments and writing for this manuscript; SK completed the proliferartion assays; MB provided guidance and completed the writing. 
